# Targeting Plasminogen Activator Inhibitor-1 with a Novel Small Molecule Inhibitor Attenuates Lung Fibrosis

**DOI:** 10.21203/rs.3.rs-6951289/v1

**Published:** 2025-08-19

**Authors:** Thomas H. Sisson, Sean Fortier, Lam C. Tsoi, Roxann Alonzo, Natalya Subbotina, Mark Warnock, Kris Mann, Sergey S. Gutor, J. Craig Hartman, Johann E. Gudjonsson, Enming J. Su, Cory D. Emal, Daniel A. Lawrence

**Affiliations:** University of Michigan; University of Michigan; University of Michigan; University of Michigan; University of Michigan; University of Michigan; University of Michigan; University of Michigan; University of Michigan; University of Michigan; University of Michigan; Eastern Michigan University; University of Michigan

## Abstract

Fibrotic lung diseases are associated with significant morbidity and mortality, and few therapies have been FDA-approved for patients with these conditions. Therefore, developing effective anti-fibrotic treatments represents an unmet clinical need. Plasminogen activator inhibitor 1 (PAI-1) is an attractive therapeutic target as its expression is up-regulated in the context of fibrotic lung disease, and a causal role for PAI-1 in lung fibrogenesis has been established in complementary animal models. Here, we study the efficacy of a novel small molecule PAI-1 inhibitor, MDI-2517, to attenuate lung fibrosis. We observed that MDI-2517 administered during the fibrotic phase of complementary murine models reduces the severity of scarring. Furthermore, we found that MDI-2517 treatment beginning on day 21 after lung injury accelerates fibrosis resolution while in vitro data reveal that this drug reverses myofibroblast differentiation. These results motivate targeting PAI-1 as a therapy for lung fibrosis and highlight MDI-2517 as a promising drug.

## Introduction

Pulmonary fibrosis is defined by the accumulation of collagen-rich matrix within the distal, alveolar regions of the lung leading to architectural distortion and physiologic dysfunction. Multiple known causes of pulmonary fibrosis have been identified, including exposure to organic and inorganic dusts, as a complication of connective tissue diseases such as rheumatoid arthritis and systemic sclerosis, and as a byproduct of medication toxicity^1^. Scarring of the lung can also occur in the absence of a known cause, and in this setting, patients are classified as having idiopathic interstitial pneumonia (IIP), with idiopathic pulmonary fibrosis (IPF) being the most common diagnosis. Diseases that result in pulmonary fibrosis are typically associated with significant morbidity and mortality, and there are currently only two FDA-approved therapies for these disorders^2^. Clinical trials have found that these treatments slow, but do not completely halt, the progression of scarring^3–5^. Therefore, new treatments that target critical profibrotic molecules and/or pathways are desperately needed.

One pro-fibrotic protein that holds promise as a therapeutic target is plasminogen activator inhibitor-1 (PAI-1). PAI-1 is a multifunctional protein with inhibitory activity against urokinase and tissue plasminogen activators (uPA and tPA) and with binding activity for the provisional matrix protein vitronectin (VTN)^6,7^. PAI-1 also binds to several cell surface receptors^8–10^, and its interaction with sortilin related receptor 1 (SorLA) is critical to its profibrotic activity^11^. A variety of interventions that modulate PAI-1 activity such as gene deletion, gene over-expression, recombinant protein reconstitution, and siRNA inhibition have established a causal relationship between this protein and the severity of lung fibrosis in complementary animal models^12–16^. Furthermore, several studies have identified PAI-1 as a critical downstream mediator of master pro-fibrotic regulators, including TGF-β and matrix stiffness^17–19^.

Based on its role in fibrosis and other diseases such as vascular thrombosis, multiple laboratories have sought to develop small molecule PAI-1 inhibitors for clinical use^20–22^. However, the structural plasticity of PAI-1 has made this process challenging^20^. One strategy has been to generate small peptides that mimic the reactive center loop, the protein domain that acts as a bait for uPA and t-PA^23,24^. Pseudopeptides represent another class of PAI-1 inhibitors. These drugs were designed to induce a structural rearrangement of PAI-1 that promotes a transition to latency^25^. TM5001 and TM5007 are the first described members in this class, and later modifications to improve oral bioavailability led to the development of TM5614, a drug that is currently in clinical trials in Japan for multiple indications including the treatment of severe COVID-19^26–28^ (jRCT2021210006). Although the original compounds in this class were designed to bind within the cleft between the strands of β-sheet A, experimental evidence confirming this binding site is lacking. Another PAI-1 inactivator widely tested in animal models is Tiplaxtinin, an indole derivative developed by Wyeth^29^. Extensive mechanistic studies indicate that Tiplaxtinin specifically and reversibly binds to active PAI-1, but that this PAI-1-drug interaction is disrupted by the presence of VTN^30^.

Although these inhibitors of PAI-1 have been extensively studied in models of vascular thrombosis/fibrinolysis, only a few published reports have described the efficacy of anti-PAI-1 therapy in limiting the severity of lung fibrosis. One study using TM5275 revealed a beneficial effect of pharmacologic PAI-1 inhibition when started on day 4 in a murine lung fibrosis model induced by adenoviral-mediated TGFβ expression^31^. In a second study, SK-216, a small molecule PAI-1 inhibitor with an unknown mechanism of action, was shown to modestly attenuate lung fibrosis in mice following bleomycin injury^18^. Whether other small molecule PAI-1 antagonists have more potent activity in limiting lung fibrosis, especially when started at later time points in the disease process, is unknown. Of note, despite the limited published data evaluating PAI-1 inhibition in models of lung fibrosis, a phase II trial of TM5614 for the treatment of interstitial lung disease associated with systemic sclerosis is pending (jRCT2021230022).

As indicated above the structural plasticity of PAI-1 and its interactions with multiple ligands has made the identification and development of small molecule PAI-1 inhibitors challenging. PAI-1 is a metastable protein that switches between multiple conformational states^24,32,33^, complicating the development of potent small-molecule inhibitors^20^. Furthermore, the stability of different PAI-1 conformational states is influenced by its association with other proteins, including VTN^24^. Thus, the effectiveness of an inhibitor may be strongly influenced by the local environment in which PAI-1 is expressed. Our laboratory has recently described a novel high-throughput screen that enabled the identification of MDI-2268, a compound that exhibits better activity than Tiplaxtinin in both ex vivo plasma and following in vivo administration^34–37^. In the present study, we tested the efficacy of an analog of MDI-2268, MDI-2517, in two complementary murine models of lung fibrosis, single-dose bleomycin, and targeted type 2 alveolar epithelial cell injury. Notably, MDI-2517 has improved pharmacokinetic properties compared to MDI-2268 and has recently entered phase 1 clinical studies (NCT06453824). We hypothesized that treatment with this small molecule inhibitor would mitigate lung fibrosis even when administered during later stages of experimental fibrogenesis. We show here that MDI-2517 significantly attenuated lung scarring even when started after day 10 of injury, and remarkably that treatment with MDI-2517 beginning as late as day 21 after bleomycin injury expedited fibrosis resolution. Thus, our data support that PAI-1 is an attractive therapeutic target in lung fibrosis and that MDI-2517 has significant potential to treat these challenging diseases.

## Materials and Methods

### Reagents.

MDI-2268 was synthesized as previously described^34^. MDI-2517 was from MDI-Therapeutic Novi Michigan. Tiplaxtinin was synthesized by Dr. Scott D. Larsen University of Michigan College of Pharmacy as described^29^. Nintedanib was from PKC Pharmaceuticals, (Woburn, MA). All drugs were administered by oral gavage in 0.5% methylcellulose in distilled water. Diphtheria toxin (DT) and bleomycin were sourced from Sigma Chemical (St. Louis, MO). Human recombinant PAI-1 and human vitronectin were from Innovative Research (Novi, MI). Recombinant human TGFβ (7754-BH) was purchased from R&D Systems, (Minneapolis, MN) and resuspended in filter-sterilized 1% BSA.

### Animals.

All animal experiments were performed in accordance with institutional guidelines set forth by the University Committee on the Use and Care of Animals (UCUCA). Transgenic mice expressing the human diphtheria toxin receptor (DTR) driven by the murine SPC promoter were generated in our laboratory on a C57BL/6 background^38^. C57BL/6 mice were purchased from Jackson Laboratories (Bar Harbor, ME).

### Drug Inhibitory Activity against Plasminogen Activators.

The activity of the small molecule PAI-1 inhibitors against PAI-1 was analyzed as previously described^34^. Briefly, recombinant active human PAI-1 was incubated at 2 nM for 15 min at 23°C with increasing concentrations of each compound in assay buffer (40 mM HEPES, pH 7.4, 100 mM NaCl, 0.005% Tween 20, 0.1% DMSO), or in assay buffer contain 50 nM vitronectin or 10% human PAI-1-depleted plasma followed by the addition of uPA (Innovative Research), and further incubated for 30 min at 23°C. At each drug concentration, parallel control reactions without PAI-1 were assembled. Residual enzymatic activity was determined by addition of an equal volume of 100 μM Z-Gly-Gly-Arg-AMC (Calbiochem) fluorogenic uPA substrate, and the rate of AMC release monitored at 23°C (excitation 370 nm and emission 440 nm). The percent change in PAI-1 activity was determined according to Eq. 1: [(E_i_ – P_i_) / E_i_] / [(E_0_ - P_0_) / E_0_], where E_i_ is the enzyme activity at drug concentration i; P_i_ is the enzyme activity in the presence of PAI-1 at drug concentration i; E_0_ is the enzyme activity in the absence of drug; and P_0_ is the enzyme activity in the presence of PAI-1 but in the absence of drug. Data were then plotted as the residual PAI-1 activity as a percentage of the control PAI-1 activity vs. compound concentration and from these curves the IC_50_ of each compound was calculated.

### Drug Pharmacokinetics.

A non-GLP comparative, single dose PK analysis of MDI-2268 versus MDI-2517 was performed by the University of Michigan Pharmacokinetics Core in CD-1 mice. Briefly, MDI-2268 or MDI-2517 were suspended in 0.5% methylcellulose in water and given orally by gavage at 30 mg/kg (10mL/kg) to 3 mice each. Blood was collected by using heparinized calibrated pipettes at 0.5h, 2h, 4h, and 7h and centrifuged immediately at 15,000g for 10 min. The plasma was collected, which was frozen at −80°C for later LC-MS analysis.

A second GLP pharmacokinetics evaluation with MDI-2517 was also performed in CD-1 mice by Charles River Laboratories and Aliri Bioanalysis (Mattawan, MI and Colorado Springs, CO, respectively). Mice were administered MDI-2517 (70, 200, 400 mg/kg/day (6.6–10 mL/kg)), for 28 days. On day 28, blood was collected pre-dose, and 0.5, 1, 2, 4, 8, 24 hr post-dose. Blood was processed to plasma similar to the above, and analyzed for parent MDI-2517 by LC-MS/MS.

### Diphtheria Toxin (DT) Administration.

Weight and age-matched wild type and diphtheria toxin receptor (DTR)-expressing mice were intraperitoneally injected with DT (Sigma Chemical, St. Louis, MO) once daily for 14 days at a dose of 12.5 μg/kg^38^. Control mice were injected for the same duration with 100 μl of PBS alone. Mice were intermittently weighed through day 21.

### Bleomycin Administration.

Weight and age matched mice were anesthetized with isoflurane and received an oropharyngeal instillation of bleomycin (2.5 u/kg in 50 μL of sterile PBS) (Sigma Pharmaceuticals). Control mice received 50 μl of PBS alone. Mice were intermittently weighed through day 21.

### Hydroxyproline assay.

Hydroxyproline content of the lung was measured as previously described^13^.

### Lung histology.

The left lung was inflation-fixed at 25 cm H_2_O pressure with 10% neutral-buffered formalin, removed en bloc, further fixed in 10% neutral-buffered formalin overnight, and then paraffin embedded. Five-micron sections were stained using hematoxylin and eosin, and picrosirius red methods.

### Bronchoalveolar lavage.

BAL fluid was generated by instilling 1.0 ml of sterile PBS via a blunted 18-gauge needle into the trachea. Recovery of the fluid was consistently 70–80% of the total instilled volume. The BAL fluid was then centrifuged at 4000 g for 10 minutes, the supernatant was removed, and the samples were stored immediately at −80° C.

### BAL Fluid PAI-1, MMP-9, and TGFβ Measurements.

Bleomycin-injured wild-type mice were treated with MDI-2517 (60 mg/kg daily) or vehicle beginning on day 11. On day 15, BAL fluid was collected, and endogenous active murine PAI-1 concentrations were measured using a Magnetic microsphere-based ELISA (Luminex) as previously described^15^. Murine MMP-9 was measured using the Luminex Mouse Magnetic Assay (R & D Systems) and TGF-b using the Milliplex MAP TGFß1 magnetic bead single plex Kit (Millipore).

### Myofibroblast reversal assay

CCL210 normal adult human lung fibroblasts (American Type Culture Collection) were cultured in low glucose DMEM (Invitrogen) supplemented with 10% FBS (Hyclone), 100 units/mL penicillin, and 100 μg/mL streptomycin (both from Invitrogen). Cells were serum starved in FBS-free DMEM overnight, and differentiation to MFs was induced by treatment with TGFβ (2ng/ml) for 48 h. TGFβ–elicited myofibroblastss were then treated for specified time points with MDI-2517 (100μM) or vehicle and analyzed via qPCR (*ACTA2* and *Col1A1*), Western blot (αSMA and type I collagen), and immunofluorescence microscopy.

#### ACTA2 and ColA1A qPCR.

Analysis of transcript expression was performed by extracting total cellular RNA using a RNeasy kit (Qiagen). cDNA was prepared using the High-Capacity cDNA Reverse Transcription Kit (Applied Biosystems), amplified with Fast SYBR Green Master Mix, and analyzed on a StepOne real-time PCR system (Applied Biosystems). Fold changes were normalized to the expression levels of the housekeeping gene GAPDH.

#### αSMA and ColA1A Western Blot.

Cells were lysed in RIPA buffer supplemented with protease inhibitors (Roche Diagnostics, 11836153001) and a phosphatase inhibitor cocktail (EMD Biosciences, 524624 and 524625). Proteins were separated by SDS-PAGE and transferred to a nitrocellulose membrane. Membranes were subsequently blocked with 5% BSA and probed with a mouse antibody specific to αSMA (Agilent, M0851; GA611), Col1A1 (CST, 91144), or GAPDH (CST, 8884; Invitrogen MA5–15738).

#### Immunofluorescence microscopy and immunohistochemistry.

CCL210 fibroblasts were plated and cultured (as above) in single chamber slides and serum starved overnight. Fibroblast differentiation into myofibroblastss was achieved by exposure to TGFβ (2 ng/mL) for 48 h. Myofibroblasts were then treated with vehicle or MDI-2517 to elicit reversal of myofibroblast differentiation. Chamber slides were washed twice with chilled PBS, fixed with freshly prepared 4% formaldehyde for 10 min, washed with PBS, and quenched with 100 mM glycine for 15 min. Blocking and permeabilization were achieved by incubating the slides for 1 h in PBS containing 10% FBS and 0.1% Triton X-100 (Sigma-Aldrich). Fixed cells were then stained for stress fibers as previously described (PMID: 33561015) using the anti–αSMA-FITC antibody (1:500; F3777, Sigma-Aldrich).

#### RNA-seq:

Bulk RNA-seq of whole lung RNA was performed in DTR expressing mice administered DT for 14 days. On day 11, subsets of mice were treated QD with 60mg/kg MDI-2517 or vehicle by gavage through day 18, after which lungs were harvested. One lung from each mouse was processed for hydroxyproline analysis and the other lung for RNA isolation. Negative control groups included DTR expressing mice who were administered PBS from day 0–14 and treated with vehicle or MDI-2517. After adapter trimming, reads were mapped to mm10 using STAR^39^, and genes were quantified with HTSeq^40^, using GENCODE vM18. Reads were modeled with DESeq2^41^. Functional enrichment analyses using hypergeometric tests were conducted using the top 500 up/down-regulated genes.

### Statistical analysis

Data are presented as means ± standard error of the means (SEM). For statistical analysis GraphPad Prism software was used and in any experiment with only two groups, a two-tailed t test was used. For experiments with more than two groups, a two-way ANOVA was used with a Tukey’s post hoc test for multiple comparisons. Outliers in all data sets were identified using the Prism ROUT test. A *p* value of less than 0.05 was considered significant.

## Results

### In vitro activity of MDI-2517 and MDI-2268

**Figure 1:**
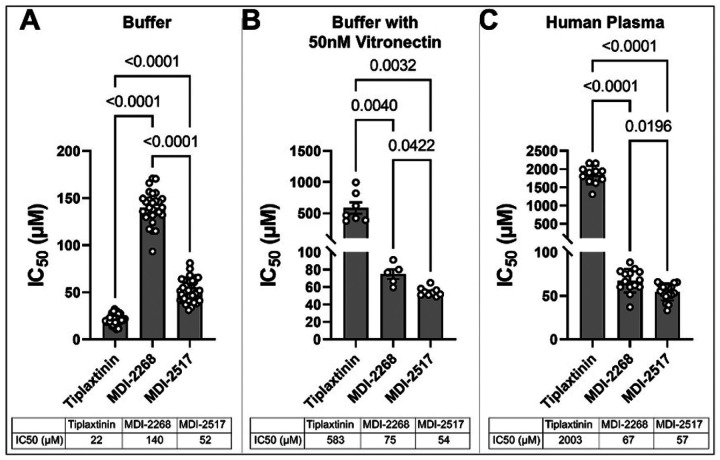
Comparison of the IC_50s_ of MDI-2517 to MDI-2268 and Tiplaxtinin. Analysis of multiple IC_50_ titrations of PAI-1 inhibition by MDI-2517, MDI-2268, and Tiplaxtinin. A) in buffer alone, B) in buffer with 50nM purified human vitronectin, C) or in buffer containing 10% human PAI-1-depleated plasma. Data is shown as mean ± SD, n is indicated in each figure by the individual data points, significance by one-way ANOVA.

To test the potency of MDI-2517, we compared increasing doses of this small molecule inhibitor to MDI-2268 and Tiplaxtinin by measuring their IC_50_ for inhibiting the activity of PAI-1 against urokinase plasminogen activator (uPA) in the presence/absence of plasma proteins. In buffer, the IC_50_ of Tiplaxtinin was lower than both MDI-2268 and MDI-2517 (Fig. 1A; IC_50_ for Tiplaxtinin = 22 μM versus 52 μM for MDI-2517 and 140 μM for MDI-2268). However, when assessed in the presence of the PAI-1 co-factor, VTN, or in human plasma, both MDI-2268 and MDI-2517 were much more efficient at inhibiting PAI-1 than Tiplaxtinin. Specifically, when assayed in the presence of VTN, both MDI-2268 and MDI-2517 were greater than 7-fold more effective than Tiplaxtinin at inhibiting PAI-1 (Fig. 1B; IC_50_ for Tiplaxtinin = 583 μM, MDI-2268 = 75 μM and MDI-2517 = 54) and this difference was even more pronounced in human plasma (Fig. 1C; IC_50_ for Tiplaxtinin = 2003 μM versus MDI-2268 = 67 μM and MDI-2517 = 57μM).

### Comparison of the pharmacokinetics of MDI-2268 and MDI-2517

**Figure 2:**
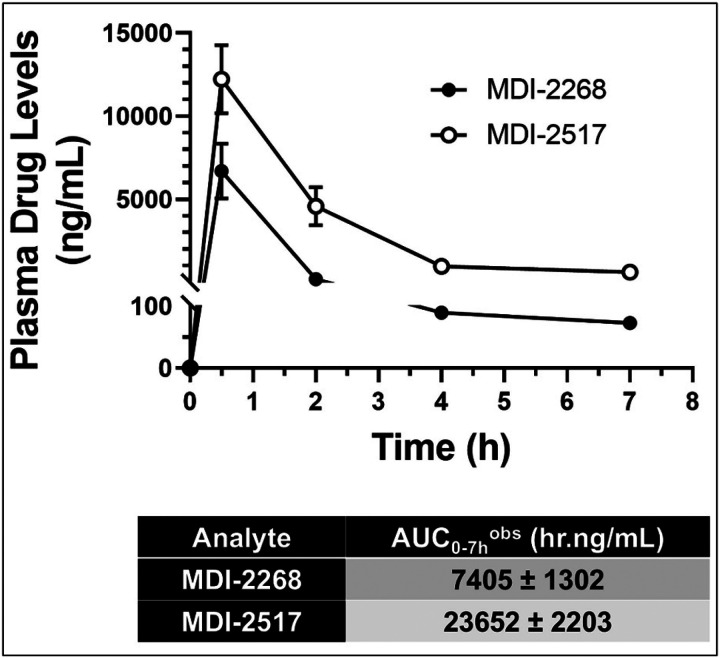
Comparison of the pharmacokinetics in mice of MDI-2517 and MDI-2268. Mice were treated with a single dose by oral gavage either MDI-2517 or MDI-2268 at 30 mg/kg. At the given time points (0.5h, 2h, 4h, and 7h), blood samples were collected in heparinized calibrated pipettes and centrifuged immediately at 15,000g for 10 min. The plasma was collected and frozen at −80°C for later analysis of the concentration of each drug by LC-MS. The data in the curves are mean at each time point ± SD and are fit with a single-phase exponential decay. The T_1/2_ and area under the curve (AUC) for each drug are shown. N=3 for each time point and each drug.

As demonstrated in Fig. 1, MDI-2268 and MDI-2517 exhibit similar efficacy in their uPA inhibitory activity. To determine if MDI-2268 and MDI-2517 have similar pharmacokinetics profiles in mice, the compounds were evaluated for pharmacokinetic characteristics by comparing single dose, oral gavage (QD) dosing. Plasma levels were measured from blood sampled sequentially up to 7 hours post-administration (Fig. 2 **and supplementary Tables 1 &2**). These results demonstrated that MDI-2517 had a 3.2-fold greater exposure, as determined for the Area Under the Curve, than MDI-2268. This higher exposure for MDI-2517 supported further nonclinical pharmacological and pharmacokinetic development of this agent.

The above study characterized the orally available pharmacokinetics of MDI-2517 as plasma levels after a single dose. We next performed a full pharmacokinetic study to more thoroughly characterize the pharmacokinetics of MDI-2517. Mice were dosed daily with MDI-2517 via oral gavage for 28 days, a duration after which MDI-2517 blood levels were at a steady state. Following the day 28 final dose, serial blood sampling was performed out to 24 hours post-dose. The results of these analyses (Table 1) demonstrate that exposure and Cmax are linear over a dose range of 70–400 mg/kg.

### Dose-response of MDI-2517 in inhibiting the development of lung fibrosis

**Figure 3:**
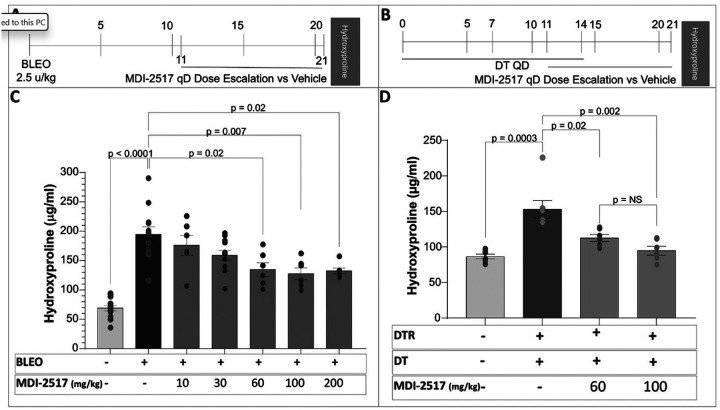
Dose-response of MDI-2517 in inhibiting the development of lung fibrosis in two complimentary lung injury models. (**A**) In the single dose bleomycin lung injury model, bleomycin was administered (2.5 u/kg in 50 μl by the oropharyngeal route) on day 0 to C57BL/6 mice. Beginning on day 11, subsets of mice were treated with daily doses of MDI-2517 (10 mg/kg to 200 mg/kg) or vehicle for 10 days. A group of uninjured C57BL/6 mice were included as a negative control. (**B**) In the targeted AEC2 injury model, diphtheria toxin (12.5 μg/kg) was administered for 14 days to DTR^+^ mice by intraperitoneal injection. Beginning on day 11, subsets of mice were treated with daily doses of MDI-2517 (60 mg/kg or 100 mg/kg) or vehicle for 10 days. A group of DTR^−^ mice treated with PBS was included as a negative control. (**C-D**) Lungs were harvested on D21 and analyzed for hydroxyproline content. n = 6–13 in single-dose bleomycin-induced injury model and n = 6–7 in targeted AEC2 injury model. Significant p values are shown from a two-way ANOVA and a Tukey’s multiple comparison test.

After determining that MDI-2517 had a better pharmacokinetics profile than MDI-2268 in mice following oral gavage administration, we focused subsequent pharmacology studies on this specific inhibitor. We first performed dose-response studies to identify the lowest effective dose of MDI-2517 in attenuating the severity of lung fibrosis in two murine models. First, in the bleomycin model, wild-type mice receive a single oropharyngeal dose of bleomycin administered on day 0 and were then treated for 10 days (beginning on day 11) with a range of once daily doses of MDI-2517 (10–200 mg/kg) or vehicle by oral gavage (Fig. 3A). At a dose of 10 mg/kg and 30 mg/kg mice showed a non-significant trend toward decrease in lung collagen content (as measured by hydroxyproline concentration). Whereas doses of 60 mg/kg, 100 mg/kg, and 200 mg/kg statistically significantly reduced day 21 lung collagen levels compared to vehicle-treated animals (Fig. 3C). In addition, these data suggest that 60 mg/kg was the maximum effective dose of MDI-2517.

We next tested MDI-2517 in a model of targeted type II alveolar epithelial cell (AEC2) injury. In this model, mice expressing the diphtheria toxin receptor (DTR) driven by the surfactant protein C promotor are administered diphtheria toxin (DT) daily for 14 days. Lung fibrosis is analyzed on day 21. To largely separate the DT-mediated injury from the antifibrotic therapeutic effects of MDI-2517, we began treatment with the PAI-1 inhibitor on Day 11 and continued through Day 21 (Fig. 3B). Informed by the results of the dose escalation in the bleomycin model, we chose to compare the efficacy of MDI-2517 at doses of 60 mg/kg and 100 mg/kg in this second model. Consistent with our prior publications, we observed an approximate 2-fold increase in lung collagen content (using hydroxyproline) in the vehicle-treated DTR-expressing mice that received 14 days of DT (Fig. 3B). The 10-day treatment course of MDI-2517 at both 60 mg/kg and 100 mg/kg doses resulted in a statistically significant attenuation in the severity of fibrosis induced by targeted AEC2 injury (Fig. 3D). As in the bleomycin model, there was no apparent difference in the efficacy of the two MDI-2517 doses, supporting that a daily 60 mg/kg dose provides maximum efficacy. Using the pharmacokinetic data in Table 1, we calculated that the maximum efficacious dose for oral administration of 60 mg/kg results in an estimated AUC_0 − 24_ of 62800 ng*h/mL (Table 2).

### Efficacy of MDI-2517 compared to Nintedanib in inhibiting lung fibrosis

**Figure 4:**
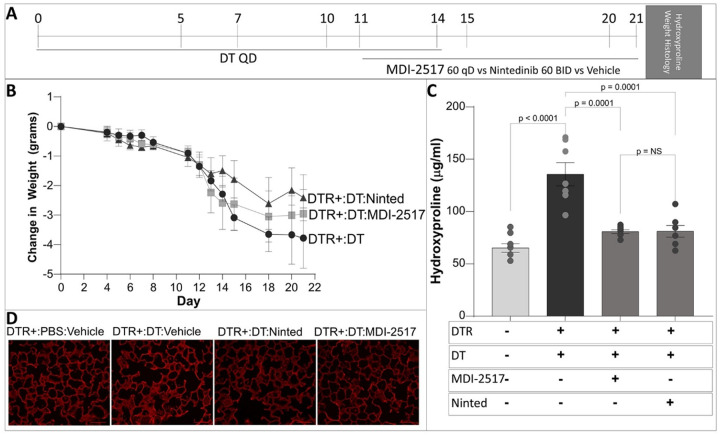
Efficacy of MDI-2517 compared to Nintedanib in inhibiting lung fibrosis following targeted AEC2 injury. (**A**) Diphtheria toxin (12.5 μg/kg) was administered for 14 days to DTR^+^ mice. Beginning on day 11, subsets of mice were treated with daily doses of MDI-2517 (60 mg/kg qD), twice daily doses of nintedanib (60 mg/kg BID) or vehicle for 10 days. A group of DTR^−^ mice treated with PBS were included as a negative control. (**B**) Mice were weighed intermittently between day 0 and day 21. Lungs were harvested on D21 and analyzed for (**C**) hydroxyproline content (n = 7–8/group) or (**D**) histopathologic changes via picrosirius red staining. Significant p values are shown from a two-way ANOVA and a Tukey’s multiple comparison test.

Nintedanib is one of two FDA-approved agents for the treatment of IPF. After establishing that the 60 mg/kg dose of MDI-2517 limits the severity of lung fibrosis in two distinct murine models, we next sought to compare the efficacy of the PAI-1 inhibitor to Nintedanib. MDI-2517 (at 60 mg/kg once daily) and Nintedanib (at 60 mg/kg twice daily) were administered for 10-days beginning on day 11 in the targeted AEC2 injury model (Fig. 4A). Endpoints included change in weight, lung hydroxyproline (quantitative biochemical measure of collagen deposition), and lung histology (a qualitative assessment of fibrosis). Consistent with prior published data, exposure of DTR-expressing mice to 14 days of DT resulted in significant weight loss (Fig. 4B). Treatment with both MDI-2517 and Nintedanib mitigated the severity of the weight loss that we observed in the vehicle-treated injured group. When comparing the two agents, we observed Nintedanib to offer a modest benefit over MDI-2517 in limiting weight loss. With respect to lung collagen accumulation, MDI-2517 and Nintedanib treatment exhibited remarkably similar efficacy, and both agents statistically significantly reduced the lung hydroxyproline content compared DRT-expressing mice injured with DT that received no treatment (Fig. 4C). Histopathologic analysis with picrosirius red staining revealed targeted AEC2 injury to result in diffuse thickening of alveolar walls and increased picrosirius red staining, supporting a deposition of collagen (Fig. 4D). Treatment with both MDI-2517 and Nintedanib significantly attenuated these changes, and there was no appreciable difference between the two agents except that MDI-2517 was dosed once a day and Nintedanib was dosed twice a day.

**Figure 5:**
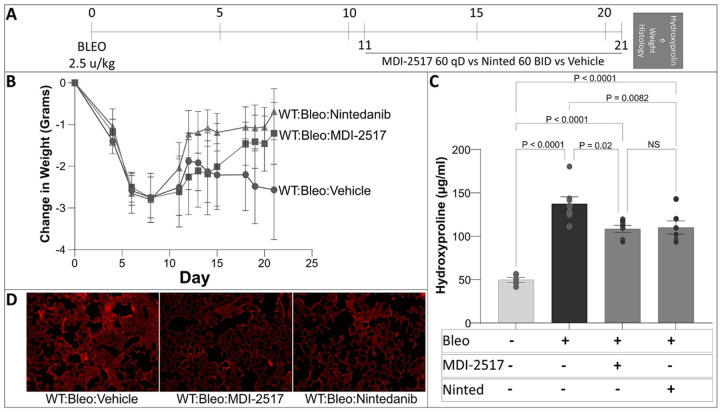
Efficacy of MDI-2517 compared to Nintedanib in inhibiting lung fibrosis following single-dose bleomycin-induced lung injury. (**A**) Bleomycin was administered (2.5 u/kg in 50 μl by the oropharyngeal route) on day 0 to C57BL/6 mice. Beginning on day 11, subsets of mice were treated with daily doses of MDI-2517 (60 mg/kg qD), twice daily doses of nintedanib (60 mg/kg BID) or vehicle for 10 days. A group of uninjured C57BL/6 mice were included as a negative control. (**B**) Mice were weighed intermittently between day 0 and day 21. (**C**) Lungs were harvested on D21 and analyzed for hydroxyproline content (n = 5–7 per group) or (**D**) histopathologic changes via picrosirius red staining. Significant p values are shown froma two-way ANOVA and a Tukey’s multiple comparison test.

We next compared the efficacy of MDI-2517 (60 mg/kg once daily) and Nintedanib (60 mg/kg twice daily) in the single-dose bleomycin model (Fig. 5A). Endpoints again included change in weight, lung hydroxyproline, and lung histology. We found that the initiation of both treatments at day 11 resulted in a recovery of body weight compared to mice treated with vehicle (Fig. 5B). Although the improvement in weight was more rapid with Nintedanib, this group had already achieved a slightly higher weight before the start of treatment. In addition to recovery of lost weight, both MDI-2517 and Nintedanib statistically significantly attenuated the lung collagen content (as measured by hydroxyproline concentration), and there was no difference in the efficacy of the two agents with respect to this endpoint, indicating that MDI-2517 was as effective as Nintedanib but with once a day dosing instead of twice a day as recommend for Nintedanib (Fig. 5C). Assessment of lung histopathology using picrosirius red staining revealed large areas of lung consolidation (comprised of increased cellular infiltrates and the accumulation of red-staining extracellular matrix) in the bleomycin-injured vehicle-treated group. In contrast, treatment with both MDI-2517 and Nintedanib significantly attenuated these regions (Fig. 5D).

### MDI-2517 effect on plasma biomarkers

**Figure 6:**
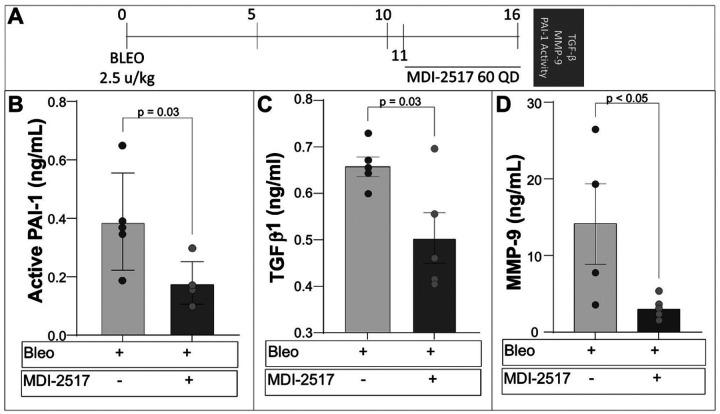
MDI-2517 treatment decreases pro-fibrotic plasma biomarkers levels following single-dose bleomycin-induced lung injury. (**A**) Bleomycin was administered (2.5 u/kg in 50 μl by the oropharyngeal route) on day 0 to C57BL/6 mice. Beginning on day 11, subsets of mice were treated with daily doses of MDI-2517 (60 mg/kg qD) or vehicle for 5 days (n = 5/group). Blood was collected for plasma preparation on day 16 and analyzed by immunologic assay for: (**B**) Active PAI-1 in plasma, (**C**) TGFβ, or (**D**) matrix metallopeptidase 9 (MMP-9). Data and error bars represent the mean ± SEM and significant p values are shown froma two-tailed Student’s t-test.

To begin interrogating the mechanism of protection afforded by MDI-2517, we measured the effect of drug treatment on bleomycin-induced biomarker levels that are known to be upregulated in human IPF patients. Mice were injured with bleomycin, and plasma was collected for biomarker analysis on day 16, 5-days after of the initiation of once daily administration of MDI-2517 (60 mg/kg) (Fig. 6A). This treatment course was chosen to capture a period of active collagen accumulation. We found that MDI-2517 administration reduced the systemic level of active PAI-1 (Fig. 6B), confirming target engagement by the drug. We also identified a reduction in the expression of key biomarkers, including TGF-β and matrix metalloproteinase-9 (MMP-9), a marker of vasculopathy^42^ that is upregulated in IPF patients^43^ (Fig. 6C, D). These results support the disease-modifying potential of MDI-2517 during that active fibrotic phase of the disease.

### MDI-2517 inhibits collagen synthetic pathways in the fibrosing lung

**Figure 7:**
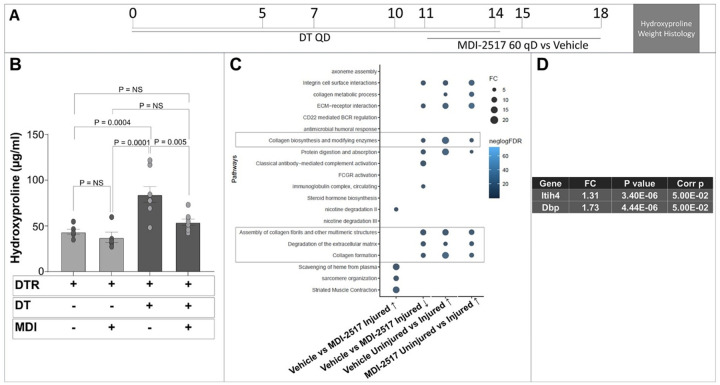
MDI-2517 treatment inhibits collagen synthetic pathways in the lung following targeted AEC2 injury. Diphtheria toxin (12.5 μg/kg) was administered for 14 days to DTR^+^ mice. Groups of PBS-administered DTR^−^ mice treated with were included as a negative control. Beginning on day 11, subsets of injured and control mice were treated with daily doses of MDI-2517 (60 mg/kg qD) or vehicle for 7 days. Lungs were harvested and one lung processed for hydroxyproline (**A**; n = 6–8/group), and one for bulk RNA seq analysis (n = 3/group). (**B**) Functional enrichment analyses using hypergeometric tests were conducted using the top 500 up/down-regulated genes. and fibrosis-associated pathways that were enriched with genes up-regulated in the AEC2 injury model were significantly enriched among genes down-regulated post MDI-2517 treatment. (**C**) Statistically significant (following multiple comparisons correction) genes upregulated by MDI-2517 versus vehicle treatment.

To further elucidate mechanistic pathways by which PAI-1 inhibition attenuates fibrosis, we performed a bulk RNAseq analysis on whole lung RNA in the targeted AEC2 injury model. For this experiment, DTR expressing mice were administered DT for 14 days. On day 11, subsets of mice were treated with MDI-2517 (60 mg/kg) or vehicle through day 18, and lungs were harvested for both hydroxyproline analysis and RNA isolation (Fig. 7A). Negative control groups included DTR expressing mice that were administered PBS from day 0–14 and treated with vehicle or MDI-2517 (60 mg/kg). Consistent with our analysis of hydroxyproline at day 21 (Fig. 4B), treatment with MDI-2517 significantly reduced lung collagen content by day 18 (Fig. 7B). Analysis of bulk RNAseq results revealed an induction of collagen synthetic pathways in response to DT-mediated injury in DTR-expressing mice. Specifically, pathways of collagen biosynthesis and modifying enzymes, collagen formation, assembly of collagen fibrils and other multimeric structures, and degradation of extracellular matrix pathways were upregulated in the vehicle-treated targeted AEC2 injury mice compared to the negative control groups (Fig. 7C). Importantly, treatment with MDI-2517 downregulated these same pathways relative to treatment with vehicle. At the individual gene level, treatment with MDI-2517 increased the expression of inter-alpha-trypsin inhibitor heavy chain 4, a protein that was found to mitigate air pollution-induced lung epithelial senescence and apoptosis (Fig. 7D). MDI-2517 also increased the expression of vitamin D binding protein (*Dbp*), and a recent review describes the potential role of vitamin D metabolism in lung fibrosis^44,45^.

### Efficacy of MDI-2517 in reversing lung fibrosis following bleomycin injury

**Figure 8:**
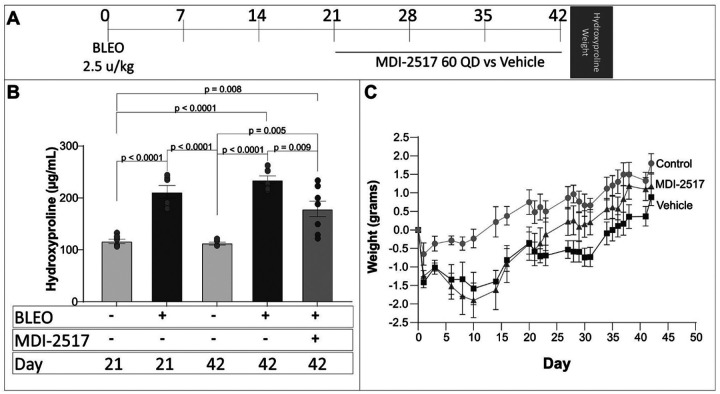
Efficacy of MDI-2517 in reversing lung fibrosis following single-dose bleomycin-induced lung injury. (**A**) Bleomycin was administered (2.5 u/kg in 50 μl by the oropharyngeal route) on day 0 to C57Bl/6 mice. Groups of uninjured C57Bl/6 mice were included as negative controls. Beginning on day 21, subsets of mice were treated with daily doses of MDI-2517 (60 mg/kg qD) or vehicle for 21 days. (**B**) Mice were weighed intermittently between day 0 and day 42. (**C**) Lungs from injured and control mice were harvested on D21 to establish pre-treatment lung collagen content and on day 42 (to establish post-treatment lung collagen content as measured by hydroxyproline concentration (n = 6–8). Significant p values are shown froma two-way ANOVA and a Tukey’s multiple comparison test.

Current FDA approved anti-fibrotic therapies have been shown to slow the rate of decline in lung function but not reverse established fibrosis. Notably, the fibrosis induced in the single-dose bleomycin injury model resolves at late time points after injury, although the rate of resolution appears to be variable between laboratories and is slowed by age^46^. Based on its ability to inhibit PAI-1 in the presence of vitronectin and other plasma proteins, we hypothesized that MDI-2517 might effectively accelerate the resolution of fibrosis during more mature stages of extracellular matrix deposition and scar formation. To test this hypothesis, we injured wild-type mice on day 0 with bleomycin and began treatment with the PAI-1 inhibitor on Day 21. Treatment was then continued daily for three weeks, at which time lung collagen content was measured using hydroxyproline (Fig. 8A). A subset of bleomycin-injured animals was analyzed for lung collagen content on Day 21 to determine the extent of fibrosis at the time of treatment initiation. The mice were also intermittently weighed over the course of the experiment. As expected, bleomycin instillation resulted in a significant increase in lung hydroxyproline at Day 21 relative to uninjured control animals (Fig. 8B). Over the ensuing 21 days, the lung hydroxyproline content in the untreated animals remained stable, indicating minimal resolution. In contrast, treatment with MDI-2517 resulted in a statistically significant reversal of lung fibrosis in the bleomycin-injured group at 42 days. This improvement in the hydroxyproline with MDI-2517 was associated with a more rapid improvement in mean body weight (Fig. 8C).

### MDI-2517 reverses in vitro myofibroblast differentiation

**Figure 9:**
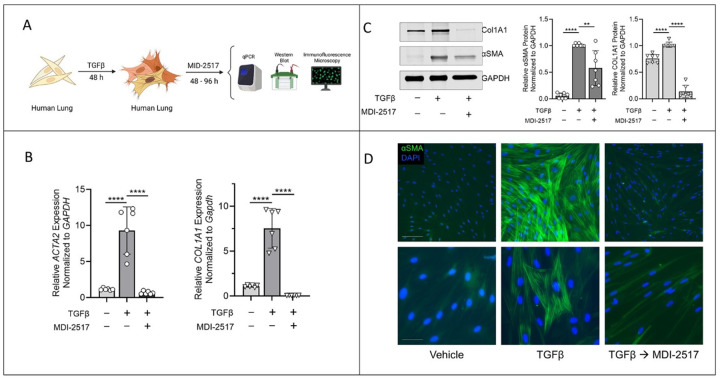
PAI-1 inhibition with MDI-2517 promotes myofibroblast dedifferentiation. (A) Schematic detailing “reversal” protocol of human lung myofibroblasts. Readouts of myofibroblast reversal following treatment with MDI-2517 (100 μM): qPCR – 48 h treatment (B), Western blot – 96 h treatment (C), and immunofluorescence microscopy of αSMA stress fibers – 96 h treatment (D) using an anti–αSMA-FITC–conjugated antibody. Nuclei were stained with DAPI. Scale bars: 20 μm (top row) and 5 μm (bottom row). The sample number (n) for experiments (B) and (C) is indicated by the number of data points in each histogram. Data and error bars represent the mean ± SEM, respectively. **P < 0.01 and ****P < 0.0001, by 2-way ANOVA.

After determining that in vivo treatment with MDI-2517 mitigated processes of collagen biosynthesis and accelerated fibrosis resolution following bleomycin-induced lung injury, we hypothesized that the mechanism of this reversal might be mediated through a drug-effect on myofibroblast phenotype. To test this hypothesis, CCL210 human lung fibroblasts were exposed to TGFβ for 48 h to induce myofibroblast differentiation, and the cultures were then treated with vehicle or 100 μM of MDI-2517 (Fig. 9A). Endpoints included αSMA and type I collagen expression (at the mRNA and protein level) and myofibroblast phenotype as determined by immunofluorescent staining of αSMA stress fiber formation. We found that treatment with the PAI-1 inhibitor for 48 h significantly reduced *ACTA2* and *Col1A1* mRNA levels back to baseline levels from a 5–10-fold increase following TGFβ exposure (Fig. 9B). Protein levels of these two myofibroblast markers were also significantly reduced by MDI-2517 after 96 h (Fig. 9C). Although αSMA protein levels did not return to baseline, immunofluorescent staining at the same time point following treatment (96 h) indicated that the incorporation of this protein into stress fibers, a hallmark of myofibroblast phenotype, was completely reversed by PAI-1 inhibition (Fig. 9D).

## Discussion

Many small molecule inhibitors of PAI-1 have been described with activity in vitro; however, the majority of these compounds display reduced efficacy in vivo. For instance, the well-studied PAI-1 inhibitor tiplaxtinin has dramatically reduced activity against vitronectin-bound PAI-1, the predominant form of PAI-1 in vivo (Fig. 1)^30^. Another reason for the lack of in vivo efficacy of some PAI-1 inhibitors is the inherent structural instability of native PAI-1, which biases high throughput screen hits toward promiscuous molecules with low affinity, hydrophobicity, and poor specificity [Reviewed in^20^]. Recently, we described a novel high-throughput screening strategy that allowed us to rapidly identify a class of PAI-1 inhibitor molecules with high potential for translation into in vivo settings. These studies led to the development of a second-generation PAI-1 inhibitor, MDI-2268, which showed significant in vivo efficacy against pathologic thrombosis^34^. Through continued medicinal chemistry, we have now generated an improved analog of MDI-2268, MDI-2517, with both more potent activity against PAI-1, and significantly improved pharmacokinetic properties. In the present study, we find that MDI-2517 can be administered orally once a day with remarkable efficacy in two different models of pulmonary fibrosis. Importantly, delayed treatment with this drug accelerated the reversal of lung scarring that occurs in the single-dose bleomycin model. Together these data suggest that MDI-2517 has considerable potential for pharmaceutical development in treating lung fibrosis and likely other diseases where excessive PAI-1 activity plays a role.

Complementary studies from different laboratories have established a causal role for PAI-1 in pulmonary fibrosis using several models of lung injury (e.g., bleomycin, TGFβ overexpression, and targeted AEC2 injury), a variety of animal species (e.g. mice and rats), and an assortment of approaches to manipulate PAI-1 activity (e.g. transgenic deficiency or over-expression of PAI-1, SiRNA inhibition, and uPA up-regulation)^47^. These data spotlight PAI-1 as an attractive therapeutic target for the mitigation of lung fibrosis, but only a few studies have interrogated the efficacy of small molecule PAI-1 inhibitors in animal models of these diseases. TM5275 was found to significantly decrease lung collagen accumulation when started 4-days after TGFβ-expressing adenovirus-induced fibrosis initiation. Although results from this study are encouraging with respect to using anti-PAI-1 agents to treat lung fibrosis, the administration of TM5275 in this study was started at an early time point. Furthermore, TGFβ is a potent inducer of PAI-1 expression, and therefore the TGFβ-overexpression model of lung fibrosis is likely to be particularly dependent on PAI-1 activity. In a second study, SK-216, a small molecule PAI-1 inhibitor with an unknown mechanism of action, was shown to attenuate lung fibrosis in mice when started on day 9 following a combination of intratracheal and oral bleomycin exposure. The ability of PAI-1 inhibition to limit lung collagen accumulation in this study when started at a delayed time point is promising. However, the improvement in scarring was modest. In the present study, we provide additional data to support the strategy of PAI-1 inhibition as a therapy for lung fibrosis. Using our novel inhibitor, MDI-2517, and two different models of lung fibrosis, we show that PAI-1 inhibition has a dose dependent effect on the severity of scarring with a maximal benefit observed at doses of 60 mg/kg and above. At 60 mg/kg, MDI-2517 significantly reduced lung collagen content to levels just above baseline and also inhibited active PAI-1 levels in the blood of the bleomycin-injured mice, supporting that its mechanism of action is, indeed, through PAI-1 antagonism.

With the mounting evidence that small molecule PAI-1 inhibitors are efficacious in animal models, it is exciting to contemplate advancing these therapies in clinical trials. Unfortunately, there is no surefire way to predict whether a drug that is efficacious in animal models in general, and the bleomycin model more specifically, will translate to patient benefit. In fact, many drugs have shown no efficacy in clinical trials. Despite this limitation, our results support the promise of PAI-1 antagonism via MDI-2517 as treatment for lung fibrosis. For example, the protective effects we observed with MDI-2517 occurred even with late onset administration of the drug (beginning at day 11 in both models). Delaying the initiation of treatment to a timepoint that is remote from the initial injury helps ensure that the intervention is working by impeding fibrogenesis rather than limiting the insult. Excitingly, we also found that MDI-2517, when started at day 21, reversed established fibrosis in the bleomycin model. Although yet to be proven, the assessment of a prospective treatment’s ability to improve rather than halt a disease process in murine models may better predict its benefits in patients who have extensive scarring at the time of diagnosis. Furthermore, the efficacy of MDI-2517 in two distinct models increases the likelihood that this drug will have efficacy in the amelioration of human disease. In fact, we have previously shown that treatment with pirfenidone and nintedanib, the only FDA-approved therapies for pulmonary fibrosis, attenuate the severity of pulmonary scarring in our model of targeted AEC2 injury. We also found that delayed treatment with several different PDE4 inhibitors reduced the severity of fibrosis in this same model, and a recent phase 3 clinical trial with BI 1015550, a preferential inhibitor of the PDE4B subtype, reduced the rate of lung function decline in patients with IPF^48^. In the present study, MDI-2517, at a lower dose and with once-a-day dosing, has equivalent efficacy to twice daily dosing of nintedanib in this same model, evidence that further supports its potential successful translation to patients.

The mechanism by which PAI-1 promotes fibrosis remains unclear. PAI-1 is a multifunctional protein with both plasminogen activator inhibitory activity and a binding affinity for non-protease ligands^7^. We recently identified sortilin related receptor 1 (SorLA), a multidomain, mosaic receptor involved in internalizing and sorting cargo proteins^49,50^, as necessary for PAI-1 to exert its profibrotic activity^11^. We also showed that SorLA and PAI-1 co-localize within cells, suggesting a previously unrecognized intercellular activity of PAI-1 in promoting fibrosis. Furthermore, in vitro studies with TM5275 and SK-216 revealed that small molecule inhibition of PAI-1 causes phenotypic alterations in both epithelial cells and fibroblasts, two key cellular constituents of parenchymal scarring. In the present study, we found that PAI-1 inhibition with MDI-2517 resulted in a dramatic reversal of myofibroblast phenotype (as measured by αSMA and type I collagen expression and stress fiber formation) in an in vitro model. This effect on myofibroblast function may explain, at least in part, the ability of MDI-2517 to accelerate the reversal of in vivo lung fibrosis. Bulk RNA seq data further indicate that PAI-1 antagonism with our drug attenuates fibrosis by downregulating pathways involved in collagen synthesis.

In regard to mechanism of action, at a more global level, we found that PAI-1 inhibition with MDI-2517 decreases plasma levels of TGF-β and MMP-9. As mentioned, TGF-β is a potent inducer of PAI-1 expression^51–53^, and PAI-1 is considered a major downstream effector of TGF-β’s profibrotic activity^54,55^. However, PAI-1 is not only induced by TGF-β; it also enhances TGF-β expression^56^ (54), potentially creating a “vicious cycle” that sustains the fibrotic response. Thus, our data support that targeting PAI-1 may directly impact the profibrotic activity of TGF-β by reducing TGF-β expression, but perhaps without the significant off-target effects of direct TGF-β inhibition. The mechanistic insight gained from the MDI-2517-driven decrease in MMP9 levels is less clear other than data from the IPF Cell Atlas indicating that MMP9 is expressed predominantly by a macrophage population that is enriched in IPF patients. This suggests that PAI-1 inhibition may also attenuate the monocyte-macrophage inflammatory process that multiple studies have shown to be critical in lung fibrogenesis, including our data in the targeted AEC2 injury model^16^.

To conclude, publications over several decades have emphasized PAI-1 as an attractive therapeutic target for the treatment of lung fibrosis, including several reports employing PAI-1 inhibitors to attenuate the severity of scarring in different animal models. Our data provide additional validation of this antifibrotic strategy and extend these prior findings by showing that 1) MDI-2517 at 60 mg/kg once daily has similar efficacy to nintedanib at 60 mg/kg twice daily, and 2) MDI-2517 accelerates the resolution of fibrosis following late-onset treatment and reverses myofibroblast differentiation. These findings, in conjunction with the drug’s effectiveness in two distinct murine models, support the development of MDI-2517 for the treatment of lung fibrosis, and its entry into phase 1 clinical studies (NCT06453824) suggest it is time to move PAI-1 antagonism from the bench to the bedside.

## Figures and Tables

**Table 1:** Pharmacokinetic Parameters of MDI-2517 in Male and Female Mouse Plasma Following Oral Administration of MDI-2517

Analyte	n / timepoint	Day	Dose (mg/kg)	C_max_ (ng/mL)	t_max_ (hr)	AUCt_last_ (hr*ng/mL)	AUC_0 – 24hr_ (hr*ng/mL)
MDI-2517	6	28	70	18600	1	65500	72100
	6		200	45900	0.5	320000	320000
	6		400	61400	0.5	560000	560000

**Table 2:** Mouse Doses and AUCs

Dose PO (gavage) (mg/kg/day)	Measured AUC_0 – 24_* (ng*h/mL)	Estimated AUC_0 – 24_** (ng*h/mL)	Duration of QD dosing (days)
60	-----	62800	10 or 21
70	72100	-----	28
200	320000	-----	28
400	560000	-----	28

* = AUC as measured bioanalytically from mouse plasma samples and derived kinetically (rows 3–5, column 2)

** = AUC as extrapolated from “Measured” AUC values (row 2, column 3)

## Data Availability

all data is included in the presented figures except for the complete set of sequence data from a bulk RNAseq experiment. This data set has been deposited in the GEO database (accession number pending).
